# Androgen deprivation in prostate cancer: benefits of home-based resistance training

**DOI:** 10.1186/s40798-020-00288-1

**Published:** 2020-12-14

**Authors:** Teresa Lam, Birinder Cheema, Amy Hayden, Stephen R. Lord, Howard Gurney, Shivanjini Gounden, Navneeta Reddy, Haleh Shahidipour, Scott Read, Glenn Stone, Mark McLean, Vita Birzniece

**Affiliations:** 1 School of Medicine, Western Sydney University, Penrith, NSW Australia; 2 Department of Diabetes and Endocrinology, Westmead Hospital, Sydney, NSW 2148 Australia; 3 Department of Diabetes and Endocrinology, Blacktown Hospital, Blacktown, NSW Australia; 4 School of Science and Health, Western Sydney University, Penrith, NSW Australia; 5 Department of Radiation Oncology, Blacktown Hospital, Blacktown, NSW Australia; 6 Crown Princess Mary Cancer Centre, Westmead Hospital, Sydney, NSW Australia; 7 NeuRA, University of New South Wales, Sydney, NSW Australia; 8 School of Medical Sciences, University of New South Wales, Sydney, NSW Australia; 9 Translational Health Research Institute, Penrith, NSW Australia; 10 School of Computing, Engineering and Mathematics, Western Sydney University, Penrith, NSW Australia; 11 Garvan Institute of Medical Research, Sydney, NSW Australia

**Keywords:** Progressive resistance training, Androgen deprivation therapy, Prostate cancer, Adverse effects

## Abstract

**Introduction:**

Androgen deprivation therapy (ADT) has detrimental effects on body composition, metabolic health, physical functioning, bone mineral density (BMD) and health-related quality of life (HRQOL) in men with prostate cancer. We investigated whether a 12-month home-based progressive resistance training (PRT) programme, instituted at the start of ADT, could prevent these adverse effects.

**Methods:**

Twenty-five patients scheduled to receive at least 12 months of ADT were randomly assigned to either usual care (UC) (*n* = 12) or PRT (*n* = 13) starting immediately after their first ADT injection. Body composition, body cell mass (BCM; a functional component of lean body mass), BMD, physical function, insulin sensitivity and HRQOL were measured at 6 weeks and 6 and 12 months. Data were analysed by a linear mixed model.

**Results:**

ADT had a negative impact on body composition, BMD, physical function, glucose metabolism and HRQOL. At 12 months, the PRT group had greater reductions in BCM by − 1.9 ± 0.8 % (*p* = 0.02) and higher gains in fat mass by 3.1 ± 1.0 % (*p* = 0.002), compared to the UC group. HRQOL domains were maintained or improved in the PRT versus UC group at 6 weeks (general health, *p* = 0.04), 6 months (vitality, *p* = 0.02; social functioning, *p* = 0.03) and 12 months (mental health, *p* = 0.01; vitality, *p* = 0.02). A significant increase in the Matsuda Index in the PRT versus UC group was noted at 6 weeks (*p* = 0.009) but this difference was not maintained at subsequent timepoints. Between-group differences favouring the PRT group were also noted for physical activity levels (step count) (*p* = 0.02). No differences in measures of BMD or physical function were detected at any time point.

**Conclusion:**

A home-based PRT programme instituted at the start of ADT may counteract detrimental changes in body composition, improve physical activity and mental health over 12 months.

**Trial registration:**

Australian and New Zealand Clinical Trials Registry, ACTRN12616001311448

**Supplementary Information:**

The online version contains supplementary material available at 10.1186/s40798-020-00288-1.

## Key points

- Androgen deprivation therapy (ADT) used in the treatment of prostate cancer has negative effects on body composition, muscle strength, insulin sensitivity and health-related quality of life (HRQOL)
- A 12-month home-based progressive resistance training (PRT) programme can offset detrimental changes in body composition, physical activity and HRQOL when initiated at the start of ADT
- A home-based PRT programme can potentially offer clinicians a viable alternative to more resource-intensive supervised programmes

## Introduction

Prostate cancer has the second highest incidence of all cancers amongst men worldwide and is the fifth leading cause of cancer death in men [1]. Androgen receptor signalling strongly promotes prostate cancer growth. Thus, androgen deprivation therapy (ADT) with gonadotrophin releasing hormone (GnRH) analogues is a commonly utilised therapy for men with prostate cancer. However, in rendering patients severely hypogonadal, ADT is associated with multiple adverse effects. Changes in body composition occur rapidly, with increases of 7–10% in fat mass (FM), and decreases of 2–4% in lean body mass (LBM) after 1 year of ADT [2], with these effects persisting up to two years following cessation [3]. Patients also experience a reduction in muscle strength and bone mineral density (BMD), increased risk of type 2 diabetes and deterioration of health-related quality of life (HRQOL) [4].

Physical exercise is currently recognised as an effective strategy to ameliorate many of the adverse effects of ADT [5]. Clinical trials have shown both resistance and aerobic exercise improve body composition, metabolic profile, functional capacity, fatigue and HRQOL [5]. Progressive resistance training (PRT) is defined as an exercise modality that involves challenging the skeletal muscles with unaccustomed loads to improve muscle mass and fitness (i.e. muscle endurance, strength and power and range of motion) over time [6]. It is well established that PRT is beneficial in the treatment of sarcopenia in older men and women [7] and is also efficacious in the treatment of ADT-induced adverse effects particularly in regards to muscle strength and body composition [8].

There are limitations relating to exercise intervention studies in prostate cancer. Firstly, much of the evidence has been derived from studies enrolling patients on stable ADT after an average treatment time of 14 months [9], while it has been shown that the development of adverse effects is the most pronounced during the initial months of ADT [10, 11]. To date, two studies have examined the benefits of a supervised exercise programme in the prevention of adverse effects when administered at the initiation of ADT. Cormie et al. [9] showed a preservation of appendicular LBM and prevention of gains in whole body FM, when compared to usual care. Using a similar intervention, Taaffe et al. [12] showed preservation of LBM and BMD after 6 months in an immediate versus delayed (waitlist) exercise groups. However, these differences became non-significant after the waitlist group completed 6 months of training (i.e. at 12 months). Secondly, studies to date have implemented supervised training programmes in exercise clinics. While compliance rates are superior compared to home-based exercise [13, 14], fully supervised programmes are resource intensive, less accessible and transitioning to community-based programmes after a period of close supervision can be confronting for some patients [15, 16]. Thus, more evidence is needed regarding the feasibility and efficacy of home-based exercise programmes. The inclusion of home-based exercise prescriptions is likely needed for the successful implementation of exercise rehabilitation as standard practice in cancer care, as recommended by the Clinical Oncological Society of Australia and exercise clinicians [17, 18].

We hypothesise that the introduction of a 12-month home-based PRT programme at the start of ADT can prevent detrimental changes in body composition, physical function, metabolic derangements and HRQOL. The primary aim of this study was to investigate the effect of PRT on body composition. As a secondary analysis, we examined the effect of PRT on physical function, BMD and HRQOL at 12 months

## Methods

**Fig. 1:**
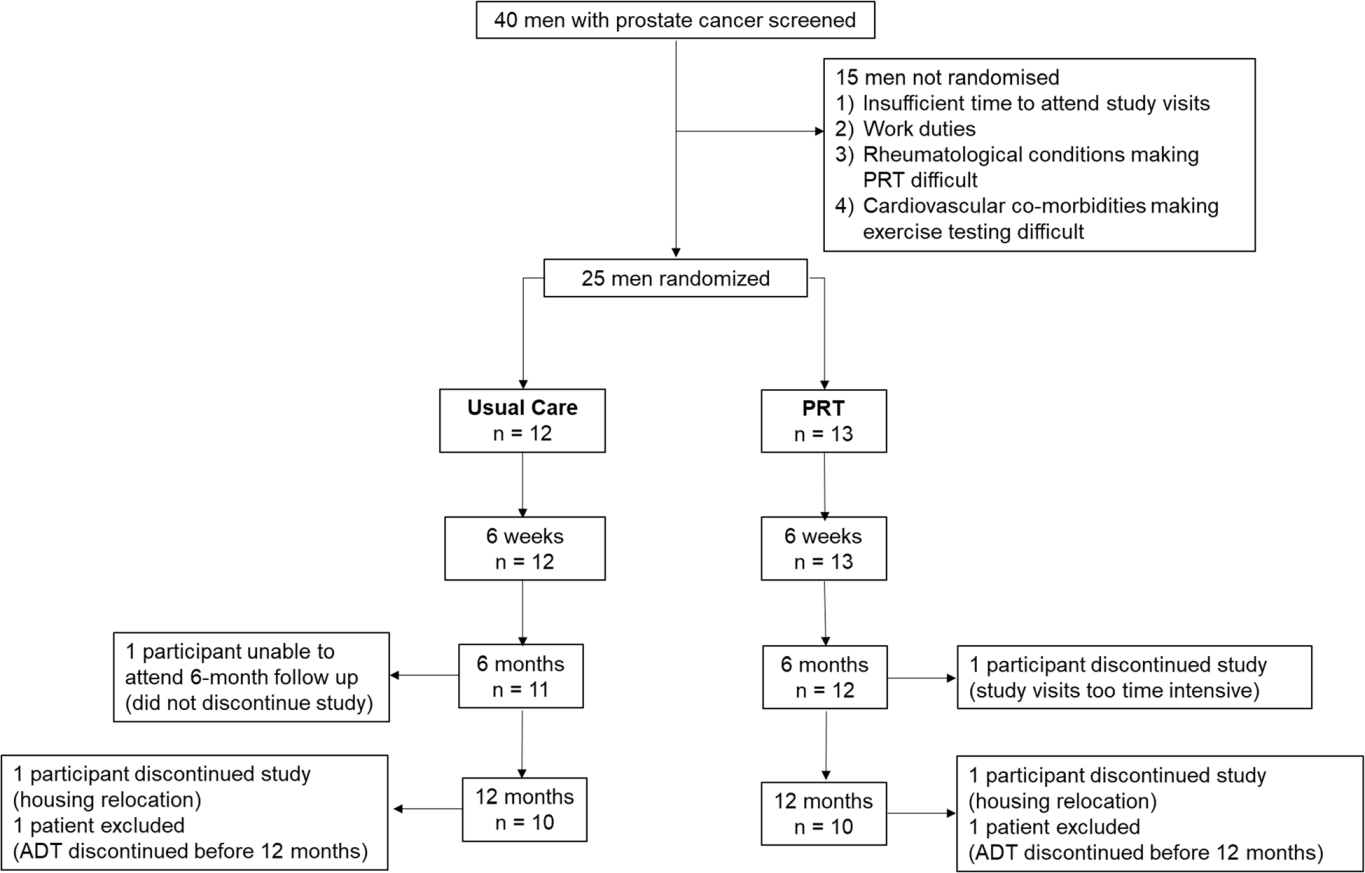
Consort diagram

This was a 12-month randomised controlled study of (PRT) in patients with prostate cancer commencing ADT. Over a period of 24 months, men with prostate cancer scheduled to receive conventional ADT with GnRH analogues were invited by their treating oncologist to participate in this study. Recruitment took place at the Crown Princess Mary Cancer Centre, Westmead Hospital and the Blacktown Cancer and Haematology Centre, Blacktown Hospital, Australia. Inclusion criteria were men aged between 50 and 80 years with histologically confirmed prostate cancer of early or locally advanced stage with ≤ 5 sites of metastases and Eastern Cooperative Oncology Group (ECOG) 0 performance status. Exclusion criteria included concurrent chemotherapy or anti-androgen therapy, previous ADT within the last 12 months, or any musculoskeletal, cardiovascular and/or neurological disorders that could inhibit them from exercising. This study was approved by the Western Sydney Local Health District Human Research Ethics Committee. The study was conducted in accordance with the principles of the Declaration of Helsinki. All participants gave written consent. The study was registered with the Australian and New Zealand Clinical Trials Registry (ACTRN12616001311448, date of registration 19th September 2016, retrospectively registered). A total of 40 patients were screened and 25 patients randomised into the study (Fig. 1).

### Experimental design

A two-armed prospective randomised controlled trial design was implemented. The 25 men were randomised into two arms: PRT (13 men) or usual care (UC) (12 men) using a computer random assignment programme. As part of routine care, all participants received non-standardised advice from the treating clinician to participate in regular exercise throughout the intervention period.

### PRT intervention

Participants assigned to the PRT group undertook 52 weeks of a home-based PRT programme starting soon after their first ADT injection. The resistance training regimen was designed to stimulate all major skeletal muscle groups and exert beneficial effects on all outcomes. The programme was designed and progressed according to standard training principles and adapted to the needs of the cohort and individual participants enrolled in the study. Resistance training was performed three times per week, with 8–10 exercises targeting the major muscle groups using adjustable dumbbells or body weight loading (callisthenics). Patients performed three sets per exercise using 8–12 repetitions maximum (RM) loading. The difficulty and/or the loading of each exercise was advanced on an individualised basis, with strength adaptation, i.e. once the participant was capable of performing greater than 12 repetitions per set [19]. There were three stages of exercises outlined in Table 1. Patients advanced to the next stage after 12 weeks, depending on their confidence in consultation with the supervising exercise physiologist.

**Table 1:** Exercise stages

Stage 1	Stage 2	Stage 3
Incline push-up	Incline push-up	Standard push-up
Bent over row	Bent over row	Decline push-up
Biceps curl	Biceps curl	Curl to press
Triceps extension	Triceps extension	Chair dips
Side shoulder raise	Side shoulder raise	Lunge
Dumbbell squat	Dumbbell squat	Side shoulder raise
Split squat	Split squat	Dumbbell squat
Straight leg deadlift	Straight leg deadlift	Straight leg deadlift
	Shoulder press	

One week of exercise supervision (two sessions) were provided at baseline to instruct patients in proper lifting techniques and loading progressions. Patients then returned for a supervised session every 12 weeks to learn the next stage of exercises and to ensure proper techniques were being utilised at home. To maximise adherence, online instructional videos and a printed training manual was provided for each exercise, and patients were given monthly reminder phone calls. Adherence to exercise was recorded in a training logbook by patients. Overall activity levels of all participants were monitored by physical activity questionnaires and total step counts for 1 week (via a pedometer) prior to their study visits.

### Endpoints

Study endpoints were assessed at baseline, 6 weeks, 6 months, and 12 months of ADT.

#### Body composition and bone mineral density

The primary endpoint was a change in LBM. LBM and total and regional FM were assessed by dual x-ray absorptiometry (DXA; GE Healthcare Lunar Prodigy Pro) and bioeletrical impedance spectroscopy (BIS) using the ImpediMed Ltd SFB7 analyser (ImpediMed Ltd Qld, Australia) [20]. Change in body cell mass (BCM), a functional component of LBM, was estimated by subtracting extracellular water (ECW) from LBM. Vertebral and hip bone mineral density was also measured by DXA at each visit.

#### Physical activity

A pedometer (G-Sensor 2026) was used to estimate step counts for 1 week prior to each visit. Patients were asked to complete an exercise diary for 1 week prior to each visit and to specify the number of hours of light, moderate or intense physical activity. Patients in the PRT group were instructed not to include the intervention in their documented activity, but to only include other types of physical activity.

#### Physical function

A series of standard tests were used to assess physical function. Maximal strength of the upper and lower body was assessed by an isometric dynamometer (i.e. triceps extension and knee extension) (Chatillon CSD200, JLW Instruments, Chicago, USA) hand grip strength by a dynamometer (Jamar Plus digital dynamometer). The best performance over 3 trials was recorded. Function lower extremity strength was assessed using the sit-to-stand test [21]. The participant is encouraged to complete as many full stands from a chair as possible within 30 s. The timed get-up-and-go test (TUGT) was used to evaluate dynamic balance and physical performance [22]. From a seated position, the participants stood, walked 3 m, turned around, walked back to the chair, returning to a seated position as quickly as possible (best performance out of three recorded). The Lord sway-meter was used to assess co-ordinated stability, which measures participants’ ability to adjust balance in a steady and co-ordinated way while placing them near or at the limits of their base of support. This test uses a 40-cm rod attached to the participant at waist level by a firm belt. Participants are then asked to adjust balance by bending or rotating their body without moving their feet, so that a pen mounted vertically at the end of the rod remains within a convoluted track. A total error score is calculated [23]. The postural sway test was used to assess dynamic balance. Participants stood barefoot on the floor or foam surface with open and closed eyes. The test records sway path, maximal anterior-posterior and lateral sway, measured by the Physiological Profile Assessment (PPA) Sway Path Mobile Applications (NeuRa) on an iPad attached to the top of an adjustable height table [24]. Aerobic capacity (submaximal VO_2_) was measured by a cycle ergometer (Lode Corival Recumbent Ergometer) and data analysed using LEM software.

#### Glucose and insulin indices

Glucose metabolism was assessed using the oral glucose tolerance test (OGTT). Blood glucose and insulin concentrations were measured at baseline and after a 75-g glucose load at 30, 60, 90, and 120 min. Hepatic insulin resistance (the product of total area under the curve for glucose and insulin during the first 30 min) and muscle insulin sensitivity (the rate of decay of plasma glucose concentration from its peak value to its nadir divided by the mean insulin concentration) were calculated [25]. Using the OGTT results, calculations were made of the HOMA-IR, the oral disposition index [26], Matsuda index (index of insulin sensitivity) [27] and overall glucose metabolism was estimated as the incremental glucose area under the curve above fasting over 120 min [26].

#### Quality of life

HRQOL was assessed at each study visit by the Short Form 36 version 2.0 (SF-36v2) physical and psychological health survey.

### Statistical analysis

Sample size calculation was based on change in body composition as indicated by LBM. Previous research involving PRT in men receiving ADT indicated a difference in LBM of 0.8 ± 0.4 kg between groups after a 12 week PRT intervention [28, 29]. Nine participants in each group were required to achieve 80% power at an *α* level of 0.05. Based on past studies involving supervised exercise programmes, an attrition rate of at least 20 % is expected. Therefore, to ensure we had sufficient participant numbers at the end of the intervention, 25 participants were randomised to the study arms (UC group 12 men; PRT group 13 men).

The statistical analysis consisted of a *linear mixed effects model*, using a random patient effect to account for differences in baseline and fixed effects for the visit and intervention arm. In this analysis, a difference in time course behaviour between study arms corresponded to an interaction between visit and intervention. Comparisons at specific time points were made using contrasts extracted from the mixed effects model and separately using two-sample *t* tests of change in endpoint. Results are expressed as mean ± S.E.M and unadjusted *P* values were utilised. A *P* value < 0.05 was considered significant. All analysis was conducted in R version 3.6.0 (Vienna, Australia), RStudio IDE (Boston, MA) and SPSS statistics v22 (IBM corporation).

## Results

### Baseline patient characteristics and retention

At baseline, 12 were enrolled in the UC arm and 13 participants in the PRT arm. At 6 months, one participant in the UC arm was unable to attend follow-up due to hospitalisation (but remained in the study) and one participant in the PRT arm discontinued the study. At 12 months, two participants discontinued the study (one from each arm) and two patients from each arm were excluded from final analysis as ADT was discontinued for more than 3 months prior to their 12-month visit (one from each arm). Thus, at 12 months, 10 participants remained in each arm (Fig. 1).

At 12 months, 50% of patients had progressed onto stage 3 exercises, 30% remained at stage 2 and 20% remained at stage 1. Patients did not progress to the next stage of exercises if they lacked the confidence and/or the ability to do so. Out of the 13 participants, one consistently did not comply with filling in his logbook citing poor literacy as the reason, although he reported full compliance with the PRT programme. Logbook analysis showed that from baseline to 6 weeks, all participants completed 100% of the exercise sessions (3 sessions/week); from 6 weeks to 6 months, an average of 2.3 sessions/week were completed; and from 6 to 12 months, an average of 2.2 sessions/week were completed.

At baseline (Table 2), UC and PRT participants were well matched in terms of age, blood pressure, number of co-morbidities and medications, body composition and PSA levels, as well as prostate cancer grade and stage. Pre-treatment testosterone levels were significant higher in the UC group (*p* = 0.02), but both groups had similar suppressed testosterone levels during ADT. There were no significant differences between groups in terms of baseline activity level as assessed by pedometer step counts and hours of light, moderate or intense physical activity.

**Table 2:** Baseline clinical characteristics

Variable	UC (*n* = 12)	PRT (*n* = 13)	***p*** value
Age (years)	71.8 ± 1.8	69.3 ± 2.3	**0.14**
Weight (kg)	79.9 ± 2.7	87.2 ± 4.7	**0.03**
BMI kg/m^2^	28.8 ± 1.1	29.7 ± 1.3	**0.36**
Number of co-morbidities^a^	0.9 ± 0.2	1.5 ± 0.2	**0.81**
Number of medications	1.1 ± 0.3	1.5 ± 1.1	**0.91**
SBP (mmHg)	137 ± 2	139 ± 4	**0.18**
DBP (mmHg)	74 ± 3	74 ± 1	**0.11**
Gleason score	7.6 ± 0.3	8.2 ± 0.2	**0.42**
Cancer staging			
Localised (*n*)	9	8	**0.36**
Biochemical recurrence (*n*)	3	3	
Metastatic (*n*)	0	2	
Previous radiotherapy (*n*)	8	5	**0.39**
Previous ADT (*n*)	0	2	**0.26**
Lean body mass (kg)	53.1 ± 1.2	54.5 ± 2.2	**0.16**
LBM (% body weight)	66.5 ± 1.8	63.2 ± 2.0	**0.68**
Fat mass (kg)	24.2 ± 2.1	29.9 ± 3.2	**0.12**
Extracellular water (L)	19.0 ± 0.6	19.8 ± 0.9	**0.05**
BCM (kg)	34.1 ± 1.0	34.7 ± 1.3	**0.58**
Testosterone (nmol/L)	16.1 ± 1.1	12.5 ± 0.8	**0.02**
LH (mIU/mL)	7.0 ± 0.7	6.3 ± 0.7	**0.70**
PSA (ng/mL)	10.3 ± 3.3	7.7 ± 1.3	**0.08**
Step count (number)	40172 ± 8502	28838 ± 5377	**0.14**
Light physical activity (h)	5.9 ± 1.6	7.9 ± 2.5	**0.09**
Moderate physical activity (h)	3.9 ± 1.8	3.3 ± 1.4	**0.93**
High physical activity (h)	0.1 ± 0.1	0.5 ± 0.3	**0.05**

Data are presented as mean ± S.E.M

*p* value is for UC vs PRT group; *BMI* body mass index, *LBM* lean body mass, *BCM* body cell mass, *LH* luteinizing hormone, *PSA* prostate-specific antigen

^a^Cardiovascular disease, respiratory disease, arthritis, hypertension, dyslipidemia, diabetes, osteoporosis

### Response to ADT

Following administration of ADT in the whole cohort serum testosterone levels significantly decreased from 14.2 ± 0.8 nmol/L at baseline to 0.2 ± 0.03 nmol/L (*p* < 0.0001) at 12 months. Similarly, serum PSA levels fell from 9.7 ± 1.4 ng/mL to 0.4 ± 0.2 ng/mL (*p* < 0.0001) at 12 months.

To eliminate possible PRT effect on ADT-induced effect size, the ADT effect is reported only in the UC group, described in detail in Supplementary Table 1. At 12 months, there was a significant reduction in BCM (BCM %) and increase in FM (FM %) as a percentage of total body mass (TBM) by 4.6 ± 0.6 % and 5.5 ± 0.6 % (*p* < 0.001), respectively (Table 3). Similarly, there were significant increases in fasting glucose by 0.7 ± 0.1 mmol/L (*p* < 0.001) and hepatic insulin resistance by 31.9 ± 11.3 (*p* < 0.01). There was a significant reduction in insulin sensitivity as represented by the Matsuda Index by 1.8 ± 0.5 (*p* < 0.01). BMD also significantly decreased at the lumbar spine by 0.01 ± 0.02 (*p* < 0.01) as did muscle strength, with a reduction in right hand grip by 4.2 ± 1.0 N (*p* < 0.001), left hand grip by 3.4 ± 1.1 N (*p* < 0.01) and lower limb strength by 50.9 ± 20.4 N (*p* < 0.05). There was a reduction in physical function as reflected by a prolongation in the TUGT by 0.3 ± 0.2 s (*p* = 0.04). HRQOL as measured by the SF36v2 mental component summary (MCS) score also decreased but did not reach significance at 12 months (*p* = 0.08).

**Table 3:** Effect of ADT on body composition, metabolism and physical function in the UC group at 12 months

	Baseline (*n* = 10)	12 months (*n* = 10)	***p*** value
Total mass (kg)	80.1 (3.0)	82.0 (3.0)	**0.002**
BCM (%)	43.6 (2.0)	39.0 (1.4)	**< 0.001**
FM (%)	29.4 (2.1)	34.9 (1.5)	**< 0.001**
Neck of femur BMD (left total)	1.011 (0.04)	1.001 (0.04)	**0.28**
Neck of femur BMD (right total)	1.01 (0.05)	0.99 (0.04)	**0.13**
Lumbar spine BMD	1.42 (0.06)	1.36 (0.07)	**0.005**
Fasting glucose (mmol/L)	4.7 (0.2)	5.4 (0.2)	**< 0.001**
Fasting insulin (IU/L)	7.1 (2.2)	11.7 (2.3)	**0.22**
Matsuda index	6.5 (1.0)	4.7 (0.8)	**0.002**
HOMA-IR	1.6 (0.5)	2.9 (0.7)	**0.11**
Hepatic insulin resistance	43.7 (12.5)	74.0 (22.7)	**0.008**
Muscle insulin resistance	1.0 (0.4)	1.3 (0.6)	**0.38**
Co-ordinated stability (corners)	0.5 (0.5)	0.8 (0.3)	**0.05**
Right hand grip (N)	37.5 (1.8)	33.2 (1.7)	**< 0.001**
Left hand grip (N)	34.0 (1.5)	30.9 (1.6)	**0.004**
Upper limb strength (N)	150.8 (14.1)	145.8 (11.3)	**0.35**
Lower limb strength (N)	297.2 (26.7)	239.5 (15.6)	**0.02**
Sit-to-stand test (number)	17.0 (1.3)	18 (1.5)	**0.17**
TUGT (s)	5.8 (0.3)	6.0 (0.3)	**0.04**
SF36v2 MCS score	58.4 (1.3)	55.4 (2.1)	**0.08**

Data are presented as mean ± S.E.M; *p* value represents change compared to baseline in the UC group at 12 months; *BCM* body cell mass, *FM* fat mass, *BMD* bone mineral density, *TUGT* timed get-up-and-go test, *IGF-1* insulin-like growth factor, *IGFBP-3* insulin-like growth factor binding protein 3

### Effect of PRT

#### Body composition

**Fig. 2:**
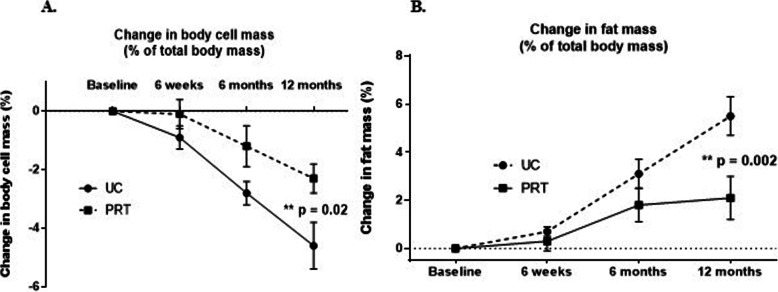
The effect of PRT on body composition. **a** Change in body cell mass (% total body mass) in the UC and PRT groups at 6 weeks and 6 and 12 months (**p = 0.01 reflects difference between the groups at 12 months). **b** Change in fat mass (% total body mass) in the UC and PRT groups at 6 weeks and 6 and 12 months (**p = 0.02 reflects difference between the groups at 12 months)

The effect of PRT on changes in body composition is shown in Fig. 2 and described in detail in Supplementary Table 2. At 6 weeks and 6 months, there was a trend towards a greater reduction in LBM % and BCM % in the UC versus PRT group but this did not reach statistical significance. At 12 months, the PRT group significantly maintained LBM by 2.7 ± 0.8 % (*p* = 0.001) and BCM by 1.9 ± 0.8 % (*p* = 0.02) (Fig. 2a) versus the UC group. Similarly, patients in the PRT group experienced less of a gain in fat mass, with a − 3.1 ± 1.0 % (*p* = 0.002) difference between the two groups at 12 months (Fig. 2b). When assessing regional fat mass, the largest difference was observed in truncal fat mass (*p* = 0.02), with the PRT group gaining 1.8 ± 0.8 kg less than the UC group at 12 months (Table 4).

**Table 4:** Group differences in the endpoints of body composition, physical activity, glucose and insulin indices and HRQOL at 6 weeks, 6 months and 12 months, reflecting the effect of PRT

Variables	6 weeks	***p*** value	6 months	***p*** value	12 months	***p*** value
Body composition						
Total mass (kg)	1.5 (0.9)	**0.09**	0.5 (0.9)	**0.60**	− 1.0 (0.9)	**0.30**
Total LBM (% total mass)	0.5 (0.8)	**0.47**	1.3 (0.8)	**0.11**	2.7 (0.8)	**0.001**
Total BCM (% total mass)	1.0 (0.8)	**0.21**	1.3 (0.8)	**0.10**	1.9 (0.8)	**0.02**
Total FM (% total mass)	− 0.4 (0.9)	**0.65**	− 1.1 (0.9)	**0.25**	− 3.1 (1.0)	**0.002**
FM trunk (kg)	− 0.1 (0.7)	**0.94**	− 0.8 (0.7)	**0.39**	− 1.8 (0.8)	**0.02**
Physical activity						
Step count	12864 (7102)	**0.08**	7719 (7308)	**0.30**	19188 (7805)	**0.02**
SF36v2 health survey						
Physical functioning	0.3 (2.6)	**0.89**	− 2.1 (2.7)	**0.45**	1.3 (2.8)	**0.63**
Role—physical	0.002 (2.1)	**0.99**	1.5 (2.2)	**0.51**	− 0.6 (2.3)	**0.81**
Bodily pain	5.0 (2.3)	**0.03**	0.3 (2.4)	**0.91**	2.6 (2.5)	**0.30**
General health	5.0 (2.4)	**0.04**	1.0 (2.6)	**0.69**	2.7 (2.6)	**0.32**
Vitality	3.8 (2.4)	**0.11**	5.8 (2.5)	**0.02**	6.0 (2.5)	**0.02**
Social functioning	2.7 (1.9)	**0.15**	4.2 (1.9)	**0.03**	2.1 (2.0)	**0.31**
Role—emotional	1.8 (2.2)	**0.43**	2.6 (2.3)	**0.28**	1.9 (2.4)	**0.43**
Mental health	2.8 (2.2)	**0.21**	3.7 (2.3)	**0.12**	4.9 (2.4)	**0.04**
Physical component summary	1.6 (1.8)	**0.40**	− 1.7 (1.9)	**0.37**	− 0.1 (2.0)	**0.97**
Mental component summary	3.4 (1.9)	**0.08**	5.7 (2.0)	**0.006**	5.5 (2.1)	**0.01**
Glucose/insulin indices						
Glucose (mmol/L) fasting	− 0.1 (0.2)	**0.73**	0.2 (0.2)	**0.34**	0.01 (0.2)	**0.94**
Insulin (IU/L) fasting	− 5.2 (3.8)	**0.18**	− 1.6 (3.9)	**0.68**	3.2 (4.1)	**0.44**
Matsuda Index	2.5 (0.8)	**0.004**	0.4 (0.8)	**0.64**	− 0.04 (0.9)	**0.96**

Data are presented as mean ± S.E.M; *p* value represents mean differences between the UC and PRT groups at 6 weeks, 6 months and 12 months; *BCM* body cell mass, *FM* fat mass

#### Physical function and BMD

A detail description of the changes in physical function and BMD is provided in Supplementary Table 3. There were no differences in BMD, muscle strength, physical function (balance and co-ordinated stability) or submaximal VO_2_ between the UC and PRT groups. However, there was a significant increase in physical activity levels as measured by step count in the PRT compared to the UC group at 12 months (*p* = 0.02) (Table 4).

#### Glucose and insulin indices

At 6 weeks, there was a decrease in the Matsuda Index in the UC group by − 0.3 ± 0.5 (*p* = 0.47) and a significant increase in the PRT group by 2.2 ± 0.7 (*p* = 0.009) with an overall difference between groups of 2.5 ± 0.8 (*p* = 0.004) (Table 4; Supplementary Table 4). However, this significant early difference was not maintained at 6 and 12 months. There were no significant differences between groups in terms of plasma insulin and glucose levels, HOMA-IR, disposition index, or liver and muscle insulin resistance.

#### Quality of life

**Fig. 3:**
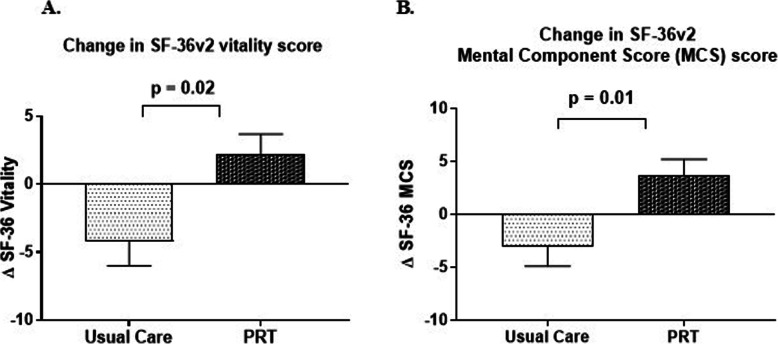
Improvements across SF-36v2 domains after 12 months of PRT. **a** Change in the SF-36v2 vitality score in the UC and PRT groups at 12 months compared to baseline (*p* = 0.02). **b** Change in SF-36v2 mental component score in the UC and PRT groups at 12 months compared to baseline (*p* = 0.01)

The effect of PRT on HRQOL is summarised in Table 4 and described in detail in Supplementary Table 5. At 6 weeks, there was a reduction in the SF36v2 general health score in the UC group and an increase in the PRT group, with a significant difference of 5.0 ± 2.4 (*p* = 0.04) between the two groups. At 6 months, there were significantly greater reductions in the SF36v2 scores for vitality (*p* = 0.02) and social functioning (*p* = 0.03) in the UC compared to the PRT group. At 12 months, the SF36v2 scores for vitality and mental health both improved in the PRT group, as opposed to a reduction in the UC group (Fig. 3a, b).

### Intervention safety and adherence

In terms of adverse events, one patient in the PRT group developed right shoulder pain (rotator cuff tendonitis) at the completion of the study, requiring physiotherapy.

## Discussion

This randomised controlled trial examined the efficacy of a 12-month home-based PRT programme in the prevention of the adverse effects of ADT. Our study demonstrated the detrimental effects of ADT on body composition, insulin resistance, BMD, physical function and HRQOL. We showed that the early implementation of a 12-month home-based PRT programme at the start of ADT resulted in beneficial effects on body composition and physical activity levels, and improvements in HRQOL.

After 12 months of ADT, we found a significant reduction in BCM (a functional component of LBM) and a significant increase in FM in the UC group. This was accompanied by a significant increase in insulin resistance and reduction in insulin sensitivity, as measured by the HOMA-IR and Matsuda index, respectively, at 6 and 12 months. As expected, there were reductions in BMD, muscle strength and physical function following 12 months of ADT.

This study showed that a home-based PRT programme was able to significantly counteract ADT-induced changes in body composition. PRT was able to offset reductions in LBM by 1.2 ± 0.2 kg at 12 months. Importantly, a similar effect of PRT was also seen with BCM, a functional component of LBM which has not been examined previously. Using a supervised PRT programme, Nilsen et al. [30] found a preservation of LBM in the PRT group, while a 12-month study conducted by Winters-Stone et al. [31] did not show any differences in LBM between the PRT and control group. However, participants in this study by Winters-Stone et al. were undergoing ADT two to three times longer than that of Nilsen et al., indicating that the benefit of exercise on LBM may diminish with longer duration of ADT [31]. Despite a lower intensity home-based programme, we were able to demonstrate a significant effect of PRT on LBM and BCM. This highlights the powerful effect of PRT on muscle, and the need for early implementation of PRT before detrimental changes in body composition occur.

Our study showed an increase in FM in both the UC and PRT groups but PRT was able to offset a gain in FM by 3.1 ± 1.0 % (2.3 ± 0.8 kg). This is similar to findings by Winters-Stone et al. [31] who found a 1.9-kg difference in FM between the UC and PRT groups after 52 weeks of supervised PRT. Importantly, we found that PRT had the greatest effect on truncal fat mass. Dickerman et al. [32] found that higher visceral fat and waist circumference, a surrogate of central adiposity, were associated with advanced and fatal prostate cancer. Thus, these results provide evidence on the benefits of home-based PRT programmes in counteracting ADT-induced negative effects on body composition in the prostate cancer population.

This study showed significant reductions in muscle strength and physical function with long-term ADT. However, there were no significant differences in muscle strength or physical function between the UC and PRT groups. Previous studies involving supervised PRT showed gains in both upper and lower limb strength in prostate cancer patients [14, 30, 33] and Taafe et al. [14] reported an improvement in physical function. It is known that home-based programmes may have lower adherence, training volume and loading compared to supervised programmes [34], which may have reduced the potential for strength adaptation or maintenance following initiation of ADT in our study.

Our study showed that overall physical activity levels were higher in the PRT compared to UC group at 12 months. Physical activity is important in prostate cancer as Phillips et al. found that a higher duration of total, non-vigorous walking activity in prostate cancer survivors was associated with improved HRQOL [35]. There is also a correlation between physical activity and reduced mortality in prostate cancer patients [36]. Thus, the finding that a home-based PRT programme can increase physical activity levels are encouraging, given the insufficient levels of physical activity in the population of men with prostate cancer, with a study by Silva et al. showing that 56.5% of a cohort of men with prostate cancer were inactive [37].

ADT results in a significant decline in bone mass and an increase in fracture risk [38, 39]. This study demonstrated a significant decline in BMD at the lumbar spine as early as 6 months of ADT, which was not attenuated by PRT. However, other parameters which would influence bone health, such as vitamin D levels, were not examined in this study and may have affected results. Our PRT programme may also have been limited in terms of the degree of impact loading which is a precipitating factor in upregulating osteoblastic activity to promote the maintenance or increase in BMD [40]. Only Winters-Stone et al. [31] has reported preservation of BMD at the L4 site in patients in the PRT group versus the control group and thus, the effect of PRT on BMD during ADT warrants further investigation.

We found an improvement in insulin sensitivity as measured by the Matsuda Index in the PRT group at 6 weeks, although this difference was not maintained at 6 and 12 months. In an RCT involving a supervised PRT programme in patients on long-term ADT, Winters-Stone et al. [31] found a non-significant reduction in insulin levels (a surrogate marker of insulin resistance) in the PRT group compared to an increase in the control group. In a population of patients with type 2 diabetes, a home-based PRT programme was also unable to maintain the glycaemic benefits obtained from supervised gymnasium-based PRT [34]. Thus, PRT may have initial benefits on glucose metabolism that is difficult to sustain during long-term ADT.

Psychological distress and anxiety is prevalent amongst men with prostate cancer ranging from 15 to 27%, and highest in those who have yet to undergo treatment [41]. This was reflected in our findings, which showed a significant decline in HRQOL following ADT in the UC group. We showed that the use of a concurrent home-based PRT programme at the start of ADT was associated with significant improvements in HRQOL. This is in line with the effects of supervised PRT programmes which showed improvements in fatigue, vitality and mental health [14, 42]. These results provide evidence that the mental health benefits of PRT are seen even when implemented in an unsupervised setting. There are multiple reasons why exercise can improve mental wellbeing. Exercise leads to improved self-esteem [43] and also induces physiological effects which impact mood and cognitive function [43]. However, it is important to note that men in the PRT group had more regular contact with study co-ordinators. This increases the participant’s social support network which can improve HRQOL [44].

There are limitations to this study. It is important to highlight that the sample size in this study was quite small, and the validity of these findings should be replicated in larger, wide-scale studies. The large number of outcome measures in relation to the small sample size raises the possibility of chance findings, although the positive findings in our study did parallel those found in previous exercise studies in a similar population. The small sample size would have affected the ability to observe differences, particularly in terms of muscle strength, physical function and the biomarkers assessed. Men in the PRT group were also relatively well-functioning individuals with minimal co-morbidities, who were highly motivated to undertake home-based exercise. Therefore, this group of participants may not be representative of men with prostate cancer at large. Men in the UC group received advice from the treating clinician regarding exercise recommendations for men with prostate cancer [45], but this was non-standardised. Study outcomes may have differed had a protocolised approach to exercise recommendations in the UC group been adopted. Furthermore, participants were asked to self-record their PRT adherence in a logbook. Although this is a frequently used adherence method, there is the possibility of inaccurate reporting with a bias towards over-reporting [46]. The use of an electronic physical activity tracker or smartphone application in future studies may reduce reporting bias [47].

This study is unique in that it explores the long-term use of isolated PRT at the start of ADT in a home-based setting which has not been investigated previously. Although there is robust data supporting the use of supervised PRT in prostate cancer patients, it is resource intensive and may not be feasible to implement across the whole prostate cancer population. We were able to demonstrate long-term positive outcomes with a more cost-effective alternative, estimated to be less than a third of the total cost of supervised programmes and hence may be more practical for the translation of exercise rehabilitation as standard practice in cancer care [17, 18]. The positive outcomes from this study suggest that guidelines can be modified to maximise exercise participation in the prostate cancer population. This involves the early involvement of an exercise physiologist to ensure proper conduct of exercises for maximal effectiveness and for periodic monitoring of exercise compliance.

## Conclusion

In conclusion, we were able to show that a home-based PRT programme instituted at the start of ADT was able to counteract detrimental changes in body composition and improve both physical activity and mental health over a 12-month period. This can potentially offer clinicians a viable alternative to more resource-intensive supervised programmes when instituted at the commencement of ADT.

## Supplementary Information

**Additional file 1: Supplementary Table 1.** Effect of ADT on body composition, metabolism, physical function and HRQOL in the UC group. **Supplementary Table 2.** Effect of PRT on body composition: absolute values and changes over 6 weeks, 6 months and 12 months. **Supplementary Table 3.** Effect of PRT on BMD, activity levels and physical function: absolute values and changes over 6 weeks, 6 months and 12 months. **Supplementary Table 4.** Effect of PRT on glucose tolerance and insulin indices: absolute values and changes over 6 weeks, 6 months and 12 months. **Supplementary Table 5.** Effect of PRT on HRQOL (SF36v2 health survey): absolute values and changes over 6 weeks, 6 months and 12 months.

## Data Availability

The datasets used and/or analysed during the current study are available from the corresponding author on reasonable request

## Abbreviations

ADT: Androgen deprivation therapy
BCM: Body cell mass
BMD: Bone mineral density
FM: Fat mass
LBM: Lean body mass
GnRH: Gonadotrophin releasing hormone
HRQOL: Health-related quality of life
PRT: Progressive resistance training
SF-36v2: Short Form 36 version 2.0
TUGT: Timed get-up-and-go test
UC: Usual care

## References

[1] Bray F, Ferlay J, Soerjomataram I, Siegel RL, Torre LA, Jemal A (2018). Global cancer statistics 2018: GLOBOCAN estimates of incidence and mortality worldwide for 36 cancers in 185 countries. CA Cancer J Clin.

[2] Galvao DA, Nosaka K, Taaffe DR, Spry N, Kristjanson LJ, McGuigan MR, Suzuki K, Yamaya K, Newton RU (2006). Resistance training and reduction of treatment side effects in prostate cancer patients. Med Sci Sports Exerc.

[3] Cheung AS, Tinson AJ, Milevski SV, Hoermann R, Zajac JD, Grossmann M (2018). Persisting adverse body composition changes 2 years after cessation of androgen deprivation therapy for localised prostate cancer. Eur J Endocrinol.

[4] Chipperfield K, Fletcher J, Millar J, Brooker J, Smith R, Frydenberg M, Burney S (2013). Predictors of depression, anxiety and quality of life in patients with prostate cancer receiving androgen deprivation therapy. Psychooncology.

[5] Kiwata JL, Dorff TB, Schroeder ET, Gross ME, Dieli-Conwright CM (2016). A review of clinical effects associated with metabolic syndrome and exercise in prostate cancer patients. Prostate Cancer Prostatic Dis.

[6] American College of Sports Medicine Position Stand (1998). Exercise and physical activity for older adults. Med Sci Sports Exerc.

[7] Peterson MD, Sen A, Gordon PM (2011). Influence of resistance exercise on lean body mass in aging adults: a meta-analysis. Med Sci Sports Exerc.

[8] Lam T, Birzniece V, McLean M, Gurney H, Hayden A, Cheema BS (2020). The adverse effects of androgen deprivation therapy in prostate cancer and the benefits and potential anti-oncogenic mechanisms of progressive resistance training. Sports Med Open.

[9] Cormie P, Galvao DA, Spry N, Joseph D, Chee R, Taaffe DR, Chambers SK, Newton RU (2015). Can supervised exercise prevent treatment toxicity in patients with prostate cancer initiating androgen-deprivation therapy: a randomised controlled trial. BJU Int.

[10] van Londen GJ, Levy ME, Perera S, Nelson JB, Greenspan SL (2008). Body composition changes during androgen deprivation therapy for prostate cancer: a 2-year prospective study. Crit Rev Oncol Hematol.

[11] Smith MR, Saad F, Egerdie B, Sieber PR, Tammela TL, Ke C, Leder BZ, Goessl C (2012). Sarcopenia during androgen-deprivation therapy for prostate cancer. J Clin Oncol.

[12] Taaffe DR, Galvão DA, Spry N, Joseph D, Chambers SK, Gardiner RA, Hayne D, Cormie P, Shum DHK, Newton RU (2019). Immediate versus delayed exercise in men initiating androgen deprivation: effects on bone density and soft tissue composition. BJU Int.

[13] Galvao DA, Newton RU, Girgis A, Lepore SJ, Stiller A, Mihalopoulos C, Gardiner RA, Taaffe DR, Occhipinti S, Chambers SK (2018). Randomized controlled trial of a peer led multimodal intervention for men with prostate cancer to increase exercise participation. Psychooncology.

[14] Taaffe DR, Newton RU, Spry N, Joseph D, Chambers SK, Gardiner RA, Wall BA, Cormie P, Bolam KA, Galvao DA (2017). Effects of different exercise modalities on fatigue in prostate cancer patients undergoing androgen deprivation therapy: a year-long randomised controlled trial. Eur Urol.

[15] Hardcastle SJ, Cohen PA (2017). Effective physical activity promotion to survivors of cancer is likely to be home based and to require oncologist participation. J Clin Oncol.

[16] Schmidt MLK, Ostergren P, Cormie P, Ragle AM, Sonksen J, Midtgaard J (2019). “Kicked out into the real world”: prostate cancer patients' experiences with transitioning from hospital-based supervised exercise to unsupervised exercise in the community. Support Care Cancer.

[17] Cheema BS, Fairman CM, Marthick M (2019). Exercise professionals in the cancer center: experiences, recommendations, and future research. Transl J Am Coll Sports Med.

[18] Cormie P, Atkinson M, Bucci L, Cust A, Eakin E, Hayes S, McCarthy S, Murnane A, Patchell S, Adams D (2018). Clinical Oncology Society of Australia position statement on exercise in cancer care. Med J Australia.

[19] Hayes SC, Spence RR, Galvao DA, Newton RU (2009). Australian Association for Exercise and Sport Science position stand: optimising cancer outcomes through exercise. J Sci Med Sport.

[20] Birzniece V, Khaw CH, Nelson AE, Meinhardt U, Ho KK (2015). A critical evaluation of bioimpedance spectroscopy analysis in estimating body composition during GH treatment: comparison with bromide dilution and dual X-ray absorptiometry. Eur J Endocrinol.

[21] Bohannon RW, Bubela DJ, Magasi SR, Wang Y-C, Gershon RC (2010). Sit-to-stand test: performance and determinants across the age-span. Isokinet Exerc Sci.

[22] Herman T, Giladi N, Hausdorff JM (2011). Properties of the 'timed up and go' test: more than meets the eye. Gerontology.

[23] Sturnieks DL, Arnold R, Lord SR (2011). Validity and reliability of the Swaymeter device for measuring postural sway. BMC geriatrics.

[24] Nr S, Nmdb R (2014). Reliability evaluation of the physiological profile assessment to assess fall risk in older people. J Gerontol Geriatr Res.

[25] Abdul-Ghani MA, Matsuda M, Balas B, DeFronzo RA (2007). Muscle and liver insulin resistance indexes derived from the oral glucose tolerance test. Diabetes Care.

[26] Utzschneider KM, Prigeon RL, Faulenbach MV, Tong J, Carr DB, Boyko EJ, Leonetti DL, McNeely MJ, Fujimoto WY, Kahn SE (2009). Oral disposition index predicts the development of future diabetes above and beyond fasting and 2-h glucose levels. Diabetes Care.

[27] Matsuda M, DeFronzo RA (1999). Insulin sensitivity indices obtained from oral glucose tolerance testing: comparison with the euglycemic insulin clamp. Diabetes Care.

[28] Dawson JK, Dorff TB, Todd Schroeder E, Lane CJ, Gross ME, Dieli-Conwright CM (2018). Impact of resistance training on body composition and metabolic syndrome variables during androgen deprivation therapy for prostate cancer: a pilot randomized controlled trial. BMC Cancer.

[29] Kiwata JL, Dorff TB, Schroeder ET, Dieli-Conwright CM (2017). Abstract 988: Effect of a supervised exercise intervention on sarcopenic obesity and metabolic syndrome in prostate cancer patients: a randomized pilot study. Cancer Research.

[30] Nilsen TS, Raastad T, Skovlund E, Courneya KS, Langberg CW, Lilleby W, Fossa SD, Thorsen L (2015). Effects of strength training on body composition, physical functioning, and quality of life in prostate cancer patients during androgen deprivation therapy. Acta oncologica.

[31] Winters-Stone KM, Dieckmann N, Maddalozzo GF, Bennett JA, Ryan CW, Beer TM (2015). Resistance exercise reduces body fat and insulin during androgen-deprivation therapy for prostate cancer. Oncol Nurs Forum.

[32] Dickerman BA, Torfadottir JE, Valdimarsdottir UA, Giovannucci E, Wilson KM, Aspelund T, Tryggvadottir L, Sigurdardottir LG, Harris TB, Launer LJ, Gudnason V, Markt SC, Mucci LA (2019). Body fat distribution on computed tomography imaging and prostate cancer risk and mortality in the AGES-Reykjavik study. Cancer.

[33] Alberga AS, Segal RJ, Reid RD, Scott CG, Sigal RJ, Khandwala F, Jaffey J, Wells GA, Kenny GP (2012). Age and androgen-deprivation therapy on exercise outcomes in men with prostate cancer. Support Care Cancer.

[34] Dunstan DW, Daly RM, Owen N, Jolley D, Vulikh E, Shaw J, Zimmet P (2005). Home-based resistance training is not sufficient to maintain improved glycemic control following supervised training in older individuals with type 2 diabetes. Diabetes Care.

[35] Phillips SM, Stampfer MJ, Chan JM, Giovannucci EL, Kenfield SA (2015). Physical activity, sedentary behavior, and health-related quality of life in prostate cancer survivors in the health professionals follow-up study. J Cancer Surviv.

[36] Bourke L, Smith D, Steed L, Hooper R, Carter A, Catto J, Albertsen PC, Tombal B, Payne HA, Rosario DJ (2016). Exercise for men with prostate cancer: a systematic review and meta-analysis. Eur Urol.

[37] Silva TD, Boing L, Dias M, Pazin J, ACdA G. Prostate cancer: quality of life and physical activity level of patients. J Phys Educ. 2018;29:e2932.

[38] Wang A, Karunasinghe N, Plank L, Zhu S, Osborne S, Bishop K, Brown C, Schwass T, Masters J, Holmes M, Huang R, Keven C, Ferguson L, Lawrenson R (2017). Effect of androgen deprivation therapy on bone mineral density in a prostate cancer cohort in New Zealand: a pilot study. Clin Med Insights Oncol.

[39] Lau YK, Lee E, Prior HJ, Lix LM, Metge CJ, Leslie WD (2009). Fracture risk in androgen deprivation therapy: a Canadian population based analysis. Can J Urol.

[40] Papadopoulos E, Mina DS, Culos-Reed N, Durbano S, Ritvo P, Sabiston CM, Krahn M, Tomlinson G, O'Neill M, Iqbal A, Timilshina N, Matthew A, Warde P, Alibhai SMH (2020). Effects of six months of aerobic and resistance training on metabolic markers and bone mineral density in older men on androgen deprivation therapy for prostate cancer. J Geriatr Oncol.

[41] Watts S, Leydon G, Birch B, Prescott P, Lai L, Eardley S, Lewith G (2014). Depression and anxiety in prostate cancer: a systematic review and meta-analysis of prevalence rates. BMJ Open.

[42] Segal RJ, Reid RD, Courneya KS, Malone SC, Parliament MB, Scott CG, Venner PM, Quinney HA, Jones LW, D'Angelo ME, Wells GA (2003). Resistance exercise in men receiving androgen deprivation therapy for prostate cancer. J Clin Oncol.

[43] Chambers SK, Dunn J, Lazenby SC, Newton RU, Cormie P, Lowe A, Sandoe D, Gardiner RA (2013). Proscare: a psychological care model for men with prostate cancer.

[44] Kollberg KS, Wilderäng U, Thorsteinsdottir T, Hugosson J, Wiklund P, Bjartell A, Carlsson S, Stranne J, Haglind E, Steineck G (2016). Psychological well-being and private and professional psychosocial support after prostate cancer surgery: a follow-up at 3, 12, and 24 months after surgery. Eur Urol Focus.

[45] Hayes S, Spence R. Prostate cancer foundation of Australia: prostate cancer and exercise. 2014. Retrieved from https://www.prostate.org.au/publications/endorsed-publications/prostate-cancer-and-exercise/.

[46] Visser M, Brychta RJ, Chen KY, Koster A (2014). Self-reported adherence to the physical activity recommendation and determinants of misperception in older adults. J Aging Phys Act.

[47] Feehan L, Geldman J, Sayre E, Park C, Ezzat A, Yoo J, Hamilton C, Li L (2018). Accuracy of Fitbit devices: a systematic review and narrative syntheses of quantitative data (Preprint). JMIR mHealth and uHealth.

