# Supplementary material for: Androgen deprivation in prostate cancer: benefits of home-based resistance training
Source: Sports Med Open. 2020 Dec 14;6:59. doi: 10.1186/s40798-020-00288-1 (PMC7736381; doi:10.1186/s40798-020-00288-1)
Supplement: Supplementary file 1 — Additional file 1: Supplementary Table 1. Effect of ADT on body composition, metabolism, physical function and HRQOL in the UC group. Supplementary Table 2. Effect of PRT on body composition: absolute values and changes over 6 weeks, 6 months and 12 months. Supplementary Table 3. Effect of PRT on BMD, activity levels and physical function: absolute values and changes over 6 weeks, 6 months and 12 months. Supplementary Table 4. Effect of PRT on glucose tolerance and insulin indices: absolute values and changes over 6 weeks, 6 months and 12 months. Supplementary Table 5. Effect of PRT on HRQOL (SF36v2 health survey): absolute values and changes over 6 weeks, 6 months and 12 months. [file 40798_2020_288_MOESM1_ESM.docx]

**Androgen deprivation in prostate cancer: benefits of home-based resistance training**

Sports Medicine Open

Teresa Lam, Birinder Cheema, Amy Hayden, Stephen R. Lord, Howard Gurney, Shivanjini Gounden, Navneeta Reddy, Haleh Shahidipour, Scott Read, Glenn Stone, Mark McLean, Vita Birzniece

**Corresponding author:**

Dr Teresa Lam

Department of Diabetes and Endocrinology

Westmead Hospital, Westmead, NSW, 2148, Australia

Phone: +61-2-8890 6796; Fax: +61-2-9635 5691

Email: [Teresa.Lam@health.nsw.gov.au](mailto:v.birzniece@westernsydney.edu.au)

**Supplementary Table 1.** Effect of ADT on body composition, metabolism, physical function and HRQOL in the UC group

| Variables | Baseline  (n = 12) | 6 weeks  (n = 12) | Change from baseline  (6 weeks) | | 6 months  (n = 11) | Change from baseline  (6 months) | | 12 months  (n = 10) | Change from baseline  (12 months) | |
| --- | --- | --- | --- | --- | --- | --- | --- | --- | --- | --- |
|  |  |  |  | **P value** |  |  | **P value** |  |  | **P value** |
| Total mass (kg) | 80.3 (2.6) | 80.0 (2.5) | -0.3 (0.5) | **0.45** | 80.7 (2.6) | 0.6 (0.5) | **0.24** | 82.0 (2.7) | 1.8 (0.5) | **0.002** |
| BCM (%) | 42.8 (1.7) | 41.9 (1.6) | -0.9 (0.6) | **0.12** | 40.7 (1.6) | -2.6 (0.6) | **<0.001** | 39.0 (1.4) | -4.6 (0.6) | **<0.001** |
| FM (%) | 30.5 (1.9) | 31.1 (1.9) | 0.7 (0.6) | **0.27** | 32.9 (1.9) | 3.0 (0.6) | **<0.001** | 34.9 (1.5) | 5.5 (0.6) | **<0.001** |
| Neck of femur BMD  (left total) | 1.002 (0.04) | 1.01 (0.04) | 0.01 (0.01) | **0.21** | 1.0002 (0.04) | 0.01 (0.01) | **0.42** | 1.001 (0.04) | -0.01 (0.01) | **0.28** |
| Neck of femur BMD  (right total) | 1.003 (0.05) | 1.015 (0.04) | 0.01 (0.01) | **0.34** | 0.9997 (0.05) | -0.0001 (0.01) | **0.95** | 0.99 (0.04) | -0.02 (0.01) | **0.13** |
| Lumbar spine BMD | 1.43 (0.05) | 1.42 (0.005) | -0.01 (0.02) | **0.53** | 1.39 (0.06) | -0.04 (0.02) | **0.03** | 1.36 (0.07) | -0.006 (0.02) | **0.005** |
| Fasting glucose (mmol/L) | 4.7 (0.1) | 4.8 (0.1) | 0.2 (0.1) | **0.19** | 5.2 (0.2) | 0.6 (0.1) | **<0.001** | 5.4 (0.2) | 0.7 (0.1) | **<0.001** |
| Fasting insulin (IU/L) | 7.2 (1.9) | 11.3 (5.6) | 4.1 (3.4) | **0.23** | 12.2 (2.3) | 4.8 (3.5) | **0.18** | 11.7 (2.3) | 4.5 (3.6) | **0.22** |
| HOMA-IR | 1.5 (0.5) | 2.5 (1.2) | 0.9 (0.8) | **0.25** | 1.3 (0.8) | 2.9 (0.6) | **0.12** | 1.4 (0.8) | 2.9 (0.7) | **0.11** |
| Matsuda index | 6.2 (0.8) | 5.8 (0.8) | -0.3 (0.5) | **0.50** | 4.3 (0.8) | -1.8 (0.5) | **0.001** | 4.7 (0.8) ` | -1.8 (0.5) | **0.002** |
| Hepatic insulin resistance | 40.7 (11.3) | 39.2 (9.3) | -1.5 (10.6) | **0.89** | 67.9 (15.4) | 27.8 (10.9) | **0.02** | 74.0 (22.7) | 31.9 (11.3) | **0.008** |
| Muscle insulin resistance | 0.9 (0.4) | 0.6 (0.3) | -0.3 (0.3) | **0.36** | 1.4 (0.6) | 0.4 (0.4) | **0.24** | 1.3 (0.6) | 0.3 (0.4) | **0.38** |
| Co-ordinated stability (corners) | 0.3 (0.2) | 0.08 (0.08) | -0.2 (0.3) | **0.55** | 0.7 (0.3) | 0.5 (0.3) | **0.09** | 0.8 (0.3) | 0.6 (0.3) | **0.05** |
| Right hand grip (N) | 37.9 (1.5) | 36.8 (1.8) | -1.1 (0.9) | **0.25** | 35.6 (1.7) | -2.6 (1.0) | **0.01** | 33.2 (1.7) | -4.2 (1.0) | **<0.001** |
| Left hand grip (N) | 34.4 (1.4) | 34.5 (1.2) | 0.1 (1.0) | **0.92** | 33.1 (1.5) | -1.5 (1.0) | **0.16** | 30.9 (1.6) | -3.4 (1.1) | **0.004** |
| Upper limb strength (N) | 156.7 (12.4) | 158.3 (11.7) | 1.6 (7.6) | **0.84** | 151.3 (9.6) | -7.2 (7.9) | **0.37** | 144.6 (11.3) ` | -7.7 (8.1) | **0.35** |
| Lower limb strength (N) | 294.2 (25.6) | 263.9 (20.8) | -30.3 (19.2) | **0.13** | 247.3 (19.2) | -46.6 (19.7) | **0.03** | 239.5 (15.6) | -50.9 (20.4) | **0.02** |
| Sit-to-stand test (number) | 17 (1.1) | 18 (1.2) | 0.8 (0.7) | **0.25** | 19 (1.7) | 1.5 (0.7) | **0.04** | 18 (1.5) | 1.0 (0.7) | **0.17** |
| TUGT (sec) | 5.6 (0.2) | 5.4 (0.2) | -0.4 (0.2) | **0.08** | 6.2 (0.3) | 0.4 (0.2) | **0.04** | 6.0 (0.3) | 0.3 (0.2) | **0.04** |
| SF36v2 MCS score | 58.2 (1.2) | 56.2 (1.8) | -2.0 (5.0) | **0.15** | 54.9 (2.5) | -2.6 (6.2) | **0.04** | 55.4 (2.1) | -3.0 (6.0) | **0.08** |

Data are presented as mean ± S.E.M; *P* value represents change compared to baseline across the cohort at 6 weeks, 6 months and 12 months; BCM, body cell mass; FM, fat mass; BMD, bone mineral density; MCS, mental component summary score

**Supplementary Table 2:** Effect of PRT on body composition: absolute values and changes over 6 weeks, 6 months and 12 months

| Variables | Baseline |  | 6 weeks |  | Group difference in mean change over 6 weeks  PRT vs UC |  | 6 months |  | Group difference in mean change over 6 mo  PRT vs UC |  | 12 months |  | Group difference in mean change over 12 mo  PRT vs UC |  |
| --- | --- | --- | --- | --- | --- | --- | --- | --- | --- | --- | --- | --- | --- | --- |
|  | UC  (n = 12) | PRT  (n = 13) | UC  (n = 12) | PRT  (n = 13) |  | **P value** | UC  (n = 11) | PRT  (n = 12) |  | **P value** | UC  (n = 10) | PRT  (n = 10) |  | **P value** |
| Total mass  (kg) | 80.3 (2.6) | 87.3 (4.6) | 80.0 (2.5) | 88.4 (4.7) | 1.5 (0.9) | **0.09** | 80.7 (2.6) | 89.3 (5.1) | 0.5 (0.9) | **0.60** | 82.0 (2.7) | 88.7 (5.5) | -1.0 (0.9) | **0.30** |
| Total LBM (kg) | 53.1 (1.2) | 54.5 (2.2) | 52.1 (1.1) | 54.7 (2.1) | 1.1 (0.7) | **0.09** | 51.4 (1.0) | 53.9 (2.2) | 1.0 (0.7) | **0.14** | 50.4 (1.2) | 52.8 (2.5) | 1.2 (0.7) | **0.10** |
| Total LBM  (% total mass) | 66.5 (1.8) | 63.2 (2.0) | 65.6 (1.7) | 62.8 (2.2) | 0.5 (0.8) | **0.47** | 64.1 (1.7) | 61.4 (2.2) | 1.3 (0.8) | **0.11** | 61.7 (1.4) | 60.4 (2.1) | 2.7 (0.8) | **0.001** |
| Total BCM (kg) | 34.1 (1.0) | 34.7 (1.3) | 33.3 (1.0) | 34.9 (1.3) | 1.0 (0.6) | **0.11** | 32.6 (1.0) | 34.1 (1.4) | 1.0 (0.7) | **0.12** | 31.8 (0.9) | 33.1 (1.6) | 0.8 (0.7) | **0.28** |
| Total BCM  (% total mass) | 42.8 (1.7) | 40.3 (1.7) | 41.9 (1.6) | 40.4 (1.8) | 1.0 (0.8) | **0.21** | 40.7 (1.6) | 38.8 (1.9) | 1.3 (0.8) | **0.10** | 39.0 (1.4) | 37.7 (2.0) | 1.9 (0.8) | **0.02** |
| Total FM (kg) | 24.1 (2.1) | 29.9 (3.2) | 24.7 (2.0) | 30.6 (3.4) | 0.2 (0.8) | **0.78** | 26.2 (2.2) | 32.3 (3.6) | -0.7 (0.8) | **0.35** | 28.5 (2.1) | 32.9 (3.6) | -2.3 (0.8) | **0.006** |
| Total FM  (% total mass) | 30.5 (1.9) | 33.9 (2.2) | 31.1 (1.9) | 34.1 (2.2) | -0.4 (0.9) | **0.65** | 32.9 (1.9) | 35.9 (2.3) | -1.1 (0.9) | **0.25** | 34.9 (1.5) | 36.1 (1.9) | -3.1 (1.0) | **0.002** |
| FM trunk (kg) | 14.8 (1.7) | 18.2 (1.9) | 15.4 (1.7) | 18.7 (2.1) | -0.1 (0.7) | **0.94** | 15.8 (1.8) | 19.1 (2.2) | -0.8 (0.7) | **0.39** | 16.8 (1.8) | 19.0 (1.9) | -1.8 (0.8) | **0.02** |
| FM android (kg) | 2.9 (0.3) | 3.4 (0.4) | 2.9 (0.4) | 3.6 (0.4) | 0.1 (0.2) | **0.67** | 3.0 (0.4) | 3.6 (0.4) | -0.1 (0.2) | **0.58** | 3.1 (0.4) | 3.5 (0.3) | -0.3 (0.2) | **0.15** |
| FM gynoid  (kg) | 3.5 (0.2) | 4.5 (0.4) | 3.6 (0.2) | 4.6 (0.5) | 0.1 (0.2) | **0.68** | 4.0 (0.2) | 4.9 (0.5) | -0.1 (0.2) | **0.51** | 4.2 (0.3) | 5.2 (0.6) | -0.03 (0.2) | **0.87** |

Data are presented as mean ± S.E.M; P value represents group differences in mean changes between PRT and UC at 6 weeks, 6 months and 12 months; LBM, lean body mass; BCM, body cell mass; FM, fat mass.

**Supplementary Table 3.** Effect of PRT on BMD, activity levels and physical function: absolute values and changes over 6 weeks, 6 months and 12 months

| Variables | Baseline |  | 6 weeks |  | Group difference in mean change at 6 weeks  PRT vs UC |  | 6 months |  | Group difference in mean change at 6 mo  PRT vs UC |  | 12 months |  | Group difference in mean change at 12 mo  PRT vs UC |  |
| --- | --- | --- | --- | --- | --- | --- | --- | --- | --- | --- | --- | --- | --- | --- |
|  | UC  (n = 12) | PRT  (n = 13) | UC  (n = 12) | PRT  (n = 13) |  | **P value** | UC  (n = 11) | PRT  (n = 12) |  | **P value** | UC  (n = 10) | PRT  (n = 10) |  | **P value** |
| Step count | 40173 (8502) | 28838 (5377) | 35166 (5647) | 36695 (6885) | 12864 (7102) | **0.08** | 35661 (7464) | 30927 (5818) | 7719 (7308) | **0.30** | 27343 (8424) | 30303 (6309) | 19188 (7805) | **0.02** |
| Physical activity (hours) |  |  |  |  |  |  |  |  |  |  |  |  |  |  |
| Light | 5.9 (1.6) | 7.9 (2.5) | 10.2 (2.7) | 9.0 (3.1) | -3.1 (2.9) | **0.28** | 7.8 (1.8) | 9.0 (2.2) | -0.6 (2.9) | **0.84** | 7.7 (2.5) | 9.9 (2.7) | -0.1 (3.2) | **0.98** |
| Moderate | 3.9 (1.8) | 3.3 (1.4) | 5.6 (2.3) | 4.2 (1.5) | -0.8 (2.0) | **0.69** | 2.7 (1.7) | 3.8 (1.5) | 1.5 (2.0) | **0.46** | 5.7 (2.3) | 2.8 (1.7) | -0.7 (2.2) | **0.74** |
| Heavy | 0.1 (0.1) | 0.5 (0.3) | 0.4 (0.3) | 1.5 (1.2) | 0.8 (0.8) | **0.32** | 0.3 (0.3) | 2.0 (1.0) | 1.1 (0.8) | **0.17** | 0.9 (0.6) | 1.4 (1.0) | 0.3 (0.9) | **0.69** |
| BMD (DXA) |  |  |  |  |  |  |  |  |  |  |  |  |  |  |
| Neck of femur (left total) | 1.002 (0.04) | 1.03 (0.04) | 1.01 (0.04) | 1.04 (0.04) | -0.004 (0.02) | **0.83** | 1.0 (0.04) | 1.02 (0.04) | -0.004 (0.02) | **0.84** | 1.0 (0.04) | 0.992 (0.04) | 0.01 (0.02) | **0.55** |
| Neck of femur  (right total) | 1.003 (0.05) | 1.003 (0.04) | 1.02 (0.04) | 1.03 (0.04) | 0.01 (0.02) | **0.45** | 1.0 (0.05) | 1.02 (0.04) | 0.02 (0.02) | **0.25** | 0.99 (0.04) | 0.995 (0.04) | 0.04 (0.02) | **0.10** |
| Spine (total) | 1.428 (0.05) | 1.22 (0.05) | 1.42 (0.05) | 1.23 (0.05) | 0.02 (0.03) | **0.58** | 1.39 (0.06) | 1.21 (0.06) | 0.03 (0.03) | **0.36** | 1.36 (0.07) | 1.18 (0.06) | 0.03 (0.03) | **0.34** |
| Physical Function |  |  |  |  |  |  |  |  |  |  |  |  |  |  |
| Balance |  |  |  |  |  |  |  |  |  |  |  |  |  |  |
| Total sway floor (EO) | 102.1 (15.0) | 96.4 (12.3) | 100.5 (18.7) | 103.0 (11.7) | 8.2 (19.2) | **0.67** | 82.5 (8.2) | 81.8 (7.8) | 0.8 (19.7) | **0.97** | 102.9 (14.7) | 100.0 (7.6) | 3.4 (20.6) | **0.87** |
| Total sway Floor (EC) | 123.7 (10.3) | 152.6 (13.5) | 137.2 (24.7) | 146.5 (11.8) | -19.6 (22.5) | **0.39** | 135.2 (10.6) | 157.6 (15.1) | -12.2 (23.1) | **0.60** | 129.9 (19.4) | 171.8 (17.3) | 7.1 (24.1) | **0.77** |
| Co-ordinated stability |  |  |  |  |  |  |  |  |  |  |  |  |  |  |
| Corners | 0.3 (0.2) | 0.3 (0.2) | 0.08 (0.08) | 0.5 (0.2) | 0.4 (0.5) | **0.42** | 0.7 (0.3) | 0.6 (0.4) | -0.2 (0.5) | **0.67** | 0.8 (0.3) | 1.7 (0.5) | 0.8 (0.5) | **0.13** |
| Sides | 1.5 (0.8) | 2.2 (0.5) | 1.3 (0.4) | 2.2 (0.6) | 0.2 (0.8) | **0.84** | 1.9 (0.5) | 1.8 (0.6) | -0.8 (1.3) | **0.37** | 1.9 (0.8) | 1.0 (0.3) | -1.7 (0.9) | **0.06** |
| Strength |  |  |  |  |  |  |  |  |  |  |  |  |  |  |
| Right hand (N) | 37.9 (1.5) | 36.3 (2.6) | 36.8 (1.8) | 36.3 (2.5) | 1.2 (1.3) | **0.37** | 35.6 (1.7) | 34.4 (2.6) | 1.1 (1.3) | **0.41** | 33.2 (1.7) | 32.2 (2.7) | 1.8 (1.4) | **0.2** |
| Left hand (N) | 34.4 (1.4) | 37.1 (2.3) | 34.5 (1.2) | 36.9 (2.5) | -0.3 (1.3) | **0.84** | 33.1 (1.5) | 34.8 (2.6) | -0.8 (1.3) | **0.57** | 30.9 (1.6) | 32.7 (3.0) | 0.2 (1.4) | **0.91** |
| Upper limb (N) | 156.7 (12.4) | 148.8 (9.0) | 158.3 (11.7) | 157.2 (8.3) | 6.8 (11.8) | **0.57** | 151.3 (9.6) | 146.3 (12.5) | 4.9 (12.2) | **0.69** | 144.6 (11.3) | 138.4 (10.0) | 1.8 (12.8) | **0.89** |
| Lower limb (N) | 294.2 (25.6) | 294.2 (19.3) | 263.9 (20.8) | 306.5 (15.3) | 42.6 (28.2) | **0.14** | 247.3 (19.2) | 258.3 (19.8) | 15.6 (28.9) | **0.59** | 239.5 (15.6) | 222.0 (23.3.3) | -11.0 (30.3) | **0.72** |
| Sit-to-Stand | 17 (1.1) | 19 (1.4) | 18 (1.2) | 21 (1.4) | 0.9 (1.0) | **0.40** | 19 (1.7) | 19 (1.2) | -0.6 (1.0) | **0.56** | 18 (1.5) | 18 (1.5) | -1.3 (1.1) | **0.25** |
| TUGT (s) | 5.6 (0.2) | 5.5 (0.3) | 5.4 (0.2) | 5.3 (0.2) | 0.2 (0.3) | **0.52** | 6.2 (0.3) | 5.5 (0.2) | -0.5 (0.3) | **0.11** | 6.0 (0.3) | 5.9 (0.2) | 0.1 (0.3) | **0.72** |
| VO_2_ max (mL/kg/min) | 26.3 (2.4) | 27.3 (1.1) | 29.1 (2.7) | 27.8 (1.8) | -2.3 (2.2) | **0.33** | 28.0 (1.7) | 25.2 (1.4) | -1.6 (2.3) | **0.60** | 29.2 (1.8) | 29.1 (1.9) | 0.9 (2.5) | **0.91** |

Data are presented as mean ± S.E.M; *P* value is for group differences in mean changes between PRT and UC at 6 weeks, 6 months and 12 months; BMD, bone-mineral density; EO, eyes opened; EC, eyes closed; TUGT, timed get-up-and-go test

**Supplementary Table 4.** Effect of PRT on glucose tolerance and insulin indices: absolute values and changes over 6 weeks, 6 months and 12 months

| Variables | Baseline |  | 6 weeks |  | Group difference in mean change over 6 weeks  PRT vs UC |  | 6 months |  | Group difference in mean change over 6 mo  PRT vs UC |  | 12 months |  | Group difference in mean change over 12 mo  PRT vs UC |  |
| --- | --- | --- | --- | --- | --- | --- | --- | --- | --- | --- | --- | --- | --- | --- |
|  | UC  (n = 12) | PRT  (n = 13) | UC  (n = 12) | PRT  (n = 13) |  | **P value** | UC  (n = 11) | PRT  (n = 12) |  | **P value** | UC  (n = 10) | PRT  (n = 10) |  | **P value** |
| Glucose (mmol/L)  Baseline | 4.7 (0.1) | 4.5 (0.1) | 4.8 (0.1) | 4.6 (0.1) | -0.1 (0.2) | **0.73** | 5.2 (0.2) | 5.3 (0.2) | 0.2 (0.2) | **0.34** | 5.4 (0.2) | 5.3 (0.2) | 0.01 (0.2) | **0.94** |
| Glucose (mmol/L)  30min | 8.3 (0.5) | 7.2 (0.4) | 7.8 (0.4) | 6.7 (0.3) | 0.1 (0.5) | **0.84** | 8.7 (0.4) | 7.7 (0.4) | 0.3 (0.5) | **0.56** | 9.0 (0.5) | 7.6 (0.4) | -0.3 (0.5) | **0.53** |
| Glucose (mmol/L)  60min | 10.3 (0.6) | 8.9 (0.5) | 9.7 (0.6) | 8.7 (0.5) | 0.4 (0.6) | **0.52** | 9.5 (0.5) | 8.5 (0.6) | 0.8 (0.6) | **0.22** | 9.4 (0.7) | 8.6 (0.5) | 0.4 (0.6) | **0.51** |
| Glucose (mmol/L)  90min | 10.4 (0.6) | 9.5 (0.5) | 9.6 (0.7) | 9.3 (0.6) | 0.6 (0.8) | **0.46** | 9.1 (0.6) | 8.2 (0.6) | 0.3 (0.8) | **0.73** | 7.4 (0.7) | 8.5 (0.5) | 1.9 (0.9) | **0.05** |
| Glucose (mmol/L)  120min | 9.3 (0.5) | 9.5 (0.5) | 8.8 (0.4) | 8.9 (0.5) | -0.1 (0.7) | **0.86** | 7.1 (0.7) | 7.5 (0.7) | 0.3 (0.8) | **0.74** | 6.7 (0.7) | 7.2 (0.5) | 0.02 (0.8) | **0.97** |
| Insulin (IU/L)  Baseline | 7.2 (1.9) | 7.9 (1.3) | 11.3 (5.6) | 6.8 (1.4) | -5.2 (3.8) | **0.18** | 12.2 (2.3) | 11.2 (1.6) | -1.6 (3.9) | **0.68** | 11.7 (2.3) | 15.8 (3.7) | 3.2 (4.1) | **0.44** |
| Insulin (IU/L)  30min | 41.8 (11.2) | 36.0 (4.9) | 37.3 (7.0) | 30.5 (6.7) | -1.0 (10.7) | **0.92** | 62.9 (13.1) | 44.7 (6.4) | -14.8 (11.1) | **0.19** | 65.9 (17.9) | 56.5 (13.7) | -3.0 (11.6) | **0.80** |
| Insulin (IU/L)  60min | 58 (14.2) | 47.3 (10.1) | 56 (13.4) | 44.7 (10.5) | -0.6 (13.9) | **0.97** | 79.7 (19.7) | 58.6 (10.9) | -9.7 (14.4) | **0.50** | 86.2 (28.2) | 66.1 (12.9) | -9.5 (15.1) | **0.53** |
| Insulin (IU/L)  90min | 79.2 (15.8) | 65.5 (9.4) | 87.3 (25.0) | 44.4 (6.9) | -29.2 (23.9) | **0.23** | 133.1 (40.2) | 63.4 (8.7) | -53.7 (24.6) | **0.03** | 73.5 (19.8) | 71.5 (14.2) | 10.8 (25.8) | **0.68** |
| Insulin (IU/L)  120min | 78.7 (22.7) | 68.5 (14.5) | 94.9 (30.8) | 46.6 (10.5) | -38.2 (26.6) | **0.15** | 115.6 (41.4) | 53.3 (10.2) | -53.6 (27.4) | **0.05** | 57.9 (10.9) | 55.1 (12.5) | -2.5 (28.6) | **0.93** |
| HOMA-IR | 1.5 (0.5) | 1.5 (0.3) | 2.5 (1.2) | 1.4 (0.3) | -1.1 (0.9) | **0.25** | 2.9 (0.6) | 2.7 (0.4) | -0.2 (0.9) | **0.83** | 2.9 (0.7) | 3.9 (1.0) | 0.9 (1.0) | **0.34** |
| Hepatic insulin resistance | 40.7 (11.3) | 32.9 (4.9) | 39.2 (9.3) | 26.9 (5.6) | -4.4 (13.1) | **0.73** | 67.9 (15.4) | 46.3 (6.5) | -14.8 (13.5) | **0.28** | 74.0 (22.7) | 62.5 (17.0) | -3.4 (14.2) | **0.81** |
| Muscle insulin resistance | 0.9 (0.4) | 0.1 (0.1) | 0.6 (0.3) | 0.1 (0.3) | 0.3 (0.4) | **0.50** | 1.4 (0.6) | 0.6 (0.2) | 0.1 (0.4) | **0.77** | 1.3 (0.6) | 0.8 (0.2) | 0.4 (0.4) | **0.38** |
| Matsuda Index | 6.2 (0.8) | 5.9 (0.7) | 5.8 (0.8) | 8.1 (1.3) | 2.5 (0.8) | **0.004** | 4.3 (0.8) | 4.6 (0.6) | 0.4 (0.8) | **0.64** | 4.7 (0.8) | 4.1 (0.8) | -0.04 (0.9) | **0.96** |
| Disposition Index | 2.6 (0.6) | 3.7 (0.7) | 3.0 (0.7) | 5.9 (1.9) | 1.7 (1.5) | **0.25** | 2.7 (0.5) | 3.4 (0.6) | -0.8 (1.50 | **0.61** | 2.9 (0.6) | 3.2 (0.9) | -0.3 (1.6) | **0.84** |

Data are presented as mean ± S.E.M; *P* value represents group differences in mean changes between PRT and UC at 6 weeks, 6 months and 12 months.

**Supplementary Table 5.** Effect of PRT on HRQOL (SF36v2 health survey): absolute values and changes over 6 weeks, 6 months and 12 months

| Variables | Baseline |  | 6 weeks |  | Group difference in mean change at 6 weeks  PRT vs UC |  | 6 months |  | Group difference in mean change at 6 mo  PRT vs UC |  | 12 months |  | Group difference in mean change at 12 mo  PRT vs UC |  |
| --- | --- | --- | --- | --- | --- | --- | --- | --- | --- | --- | --- | --- | --- | --- |
|  | UC  (n = 12) | PRT  (n = 13) | UC  (n = 12) | PRT  (n = 13) |  | **P value** | UC  (n = 11) | PRT  (n = 12) |  | **P value** | UC  (n = 10) | PRT  (n = 10) |  | **P value** |
| Physical functioning | 50.7 (2.4) | 52.3 (1.1) | 50.0 (2.6) | 52.1 (1.6) | 0.3 (2.6) | **0.89** | 50.1 (2.7) | 49.4 (2.4) | -2.1 (2.7) | **0.45** | 49.1 (3.1) | 49.7 (2.8) | 1.3 (2.8) | **0.63** |
| Role- physical | 49.7 (2.8) | 50.8 (1.4) | 51.5 (2.5) | 52.7 (4.7) | 0.002 (2.1) | **0.99** | 47.7 (3.4) | 49.1 (1.7) | 1.5 (2.2) | **0.51** | 48.4 (3.0) | 49.7 (2.3) | -0.6 (2.3) | **0.81** |
| Bodily pain | 55.8 (2.1) | 53.0 (1.6) | 54.7 (2.5) | 56.9 (1.7) | 5.0 (2.3) | **0.03** | 56.2 (2.4) | 53.6 (1.5) | 0.3 (2.4) | **0.91** | 53.8 (2.2) | 54.2 (2.4) | 2.6 (2.5) | **0.30** |
| General health | 55.0 (2.1) | 50.6 (2.2) | 54.1 (2.4) | 54.7 (2.3) | 5.0 (2.4) | **0.04** | 57.4 (2.6) | 52.8 (2.0) | 1.0 (2.6) | **0.69** | 54.4 (3.5) | 51.5 (2.0) | 2.7 (2.6) | **0.32** |
| Vitality | 59.3 (2.0) | 55.3 (1.8) | 56.6 (3.1) | 56.4 (2.3) | 3.8 (2.4) | **0.11** | 54.1 (3.2) | 54.3 (2.8) | 5.8 (2.5) | **0.02** | 54.7 (3.2) | 56.2 (1.9) | 6.0 (2.5) | **0.02** |
| Social functioning | 54.8 (1.4) | 54.9 (2.1) | 51.9 (2.4) | 54.6 (1.3) | 2.7 (1.9) | **0.15** | 50.8 (2.5) | 55.3 (1.1) | 4.2 (1.9) | **0.03** | 51.3 (2.6) | 53.3 (1.8) | 2.1 (2.0) | **0.31** |
| Role- emotional | 53.8 (1.8) | 52.8 (1.8) | 54.1 (1.7) | 54.8 (0.8) | 1.8 (2.2) | **0.43** | 51.3 (2.8) | 52.7 (1.3) | 2.6 (2.3) | **0.28** | 51.0 (3.1) | 51.6 (1.6) | 1.9 (2.4) | **0.43** |
| Mental health | 58.7 (1.2) | 54.1 (2.4) | 56.3 (1.6) | 57.4 (1.6) | 2.8 (2.2) | **0.21** | 56.8 (2.0) | 56.9 (1.9) | 3.7 (2.3) | **0.12** | 57.2 (2.1) | 50.8 (2.4) | 4.9 (2.4) | **0.04** |
| Physical component summary | 50.8 (2.4) | 51.6 (1.4) | 51.1 (2.6) | 53.4 (1.7) | 1.6 (1.8) | **0.40** | 51.3 (3.0) | 49.3 (1.9) | -1.7 (1.9) | **0.37** | 48.1 (3.3) | 49.6 (2.1) | -0.1 (2.0) | **0.97** |
| Mental component summary | 58.2 (1.2) | 54.4 (2.1) | 56.2 (1.8) | 55.9 (1.3) | 3.4 (1.9) | **0.08** | 54.9 (2.5) | 56.7 (1.8) | 5.7 (2.0) | **0.006** | 55.4 (2.1) | 56.3 (2.1) | 5.5 (2.1) | **0.01** |

Data are presented as mean ± S.E.M; *P* value represents group differences in mean changes between PRT and UC at 6 weeks, 6 months and 12 months.
